# Evaluation of altitude-appropriate reference ranges for neutrophils in diagnosis of sepsis in very low birth weight infants: A multicenter retrospective study

**DOI:** 10.1371/journal.pone.0171571

**Published:** 2017-02-09

**Authors:** Jianhui Wang, Jialin Yu, Juan Fan, Yu He, Wenhui Dong, Zhengli Wang

**Affiliations:** 1 Department of Neonatology, Children’s Hospital of Chongqing Medical University, Chongqing, China; 2 Ministry of Education Key Laboratory of Child Development and Disorders, Chongqing, China; 3 China International Science and Technology Cooperation Base of Child Development and Critical Disorders, Chongqing, China; 4 Chongqing Key Laboratory of Child Infection and Immunity, Chongqing, China; Centre Hospitalier Universitaire Vaudois, FRANCE

## Abstract

**Background:**

The circulating neutrophil count was commonly believed to be influenced by altitude. At present, neutrophil reference ranges (RRs) for very low birth weight (VLBW) neonates are only available from the sea level and from high altitude. This study aimed to construct RRs for neutrophils in VLBW infants in an intermediate-altitude area and assess its usefulness in evaluation for sepsis.

**Methods:**

This was a multicenter retrospective study of 2173 VLBW infants admitted to 20 hospitals in Chongqing in southwest of China with altitude from 1000 to 2600 feet. The RRs for absolute total neutrophils (ATN), absolute total immature neutrophils (ATI), and immature: total neutrophil proportion were constructed based on “normal” neonates (unlikely infection). Values of neutrophil from septic and uninfected neonates were respectively assessed using the revised RRs and the previous Mouzinho’s and Schmutz’s RRs. The sensitivity, specificity, positive predictive value (PPV), and negative predictive value (NPV) were compared using the McNemar’s test or *χ*^*2*^ test.

**Results:**

The upper limits for ATN and ATI using the revised RRs were much higher than those using Mouzinho’s RRs, but lower than those using Schmutz’s RRs. The revised RRs and Mouzinho’s RRs had higher sensitivities than Schmutz’s RRs at 73–672 h. The revised RRs had a higher specificity than Mouzinho’s RRs at both 0–72 h and 73–672 h. The NPV for any abnormality in neutrophil values was high at both 0–72 h and 73–672 h irrespective of the RRs used.

**Conclusion:**

Altitude-appropriate RRs for neutrophils is more suitable to guide the diagnosis and management of neonatal sepsis in VLBW infants.

## Introduction

Sepsis continues to be a leading cause of neonatal morbidity and mortality[1]. Very low birth weight (VLBW) neonates are at a higher risk for developing severe sepsis than term or near-term neonates due to insufficient innate immunity and perinatal risk factors[2]. Due to the lack of specific clinical signs, numerous investigators have evaluated the usefulness of various markers of infection in diagnosing sepsis, including circulating complete blood count (CBC), C-reactive protein (CRP), procalcitonin (PCT), and numerous other tests[3, 4]. The CBC, especially neutrophil parameters, is still one of the most widely used tests in clinic. In a survey of neonatologists’ practices, 99% obtained a CBC count when evaluating a newborn for sepsis[5]. The American Centers for Disease Control considers CBC important while deciding whether to begin antibiotics in the case of a well-appearing neonate who has risk factors for infection[6]. However, because VLBW infants commonly have other diseases that can affect neutrophil counts and their survival is relatively lower, the reference ranges (RRs) for circulating neutrophils in VLBW infants have remained undefined for a long time.

In 1994, Mouzinho and co-workers[7] reported primary RRs for neutrophil parameters in the VLBW infants, including absolute total neutrophils (ATN), absolute total immature neutrophils (ATI), and immature: total neutrophil proportion (I:T). This landmark publication established a method of determining whether the neutrophil counts of a VLBW infant should be considered normal. In 2008, Schmutz and colleagues[8] revised the RRs with a larger sample size based on a web-based electronic medical record application, suggesting a higher upper limit value of ATN than that reported by Mouzinho[7] and considering the higher altitude of the study area as a major cause for the difference. This study further confirmed a positive non-liner correlation between ATN and the altitude previously reported by Akenzua[9] Maynard[10], and Carballo[11]. In a subsequent study, Lambert et al[12] found 14 neonates who received antibiotic treatment just because of elevated ATN to be normal when retrospectively evaluated using the altitude-appropriate RRs, and concluded that neutrophil count can be termed “abnormal” only if it is evaluated using altitude-appropriate RRs.

At present, the commonly used neutrophil RRs for VLBW infants are available from the sea level reported by Mouzinho[7], and from altitudes of 4000–5200 feet reported by Schmutz[8]. The purpose of this study was to describe the RRs for VLBW infants’ neutrophils in an district with intermediate-altitude. We also assessed the usefulness of altitude appropriate RRs in diagnosis of sepsis in VLBW infants.

## Patients and methods

### Study population

This was a multicenter retrospective study of VLBW (<1500 g birth weight) infants who were admitted to 20 hospitals from April 2008 to March 2016. The 20 hospitals included all level II and level III neonatal intensive care units (NICU) in Chongqing in southwest of China with altitude from 1000 to 2600 feet, and all the hospitals were registered in the Chinese Critical Neonatal Network (CNEONET, http://www.cneonet.cn). All data in this study were collected from the CNEONET. The network maintained a standardized NICU database including demographic and clinical information, such as neonatal birth weight, gender, intrapartum and delivery room variables, maternal and neonatal complications, laboratory results, bacterial culture results, and diagnosis. The data release was managed by the management committee of CNEONET. Inclusion criterion in this study was the VLBW infants undergoing a comprehensive evaluation for sepsis or infection on the basis of risk factors or clinical signs, which consisted of CBCs, CRP, PCT, and two blood cultures collected from different sites. Neonates with congenital malformations and those who had no available information or no CBC tested were excluded. This study was approved by the Institutional Review Board of Children’s Hospital of Chongqing Medical University (Approval No.84/2016). And this study was registered in the Chinese Clinical Trial Registry (No. ChiCTR-EOC-16008790).

### Laboratory methods

CBCs were determined in all neonates at the time of evaluation for sepsis. Peripheral blood nucleated cell counts were calculated using automated hematology analyzers, and a manual 100-cell differential cell count was performed on Wright-stained blood films. Neutrophil parameters including ATN, ATI, and I:T were calculated as previously described by Manroe[13] and Mouzinho[7]. Blood cultures and CRP and PCT samples were obtained simultaneously with sampling for the initial CBC, ahead of administering antibiotics. Sequential CBC values were available for analysis 48–72 h after the initial CBC.

### Diagnosis of sepsis

Sepsis was categorized as early-onset sepsis (EOS) when the diagnosis was performed in the first 72 h of life and late-onset sepsis (LOS) when the diagnosis was carried out beyond 72 h. The likelihood of sepsis was assessed based on culture results, perinatal risk factors, clinical signs, and laboratory test results[4]: i. proven sepsis (positive blood cultures; in the case of coagulase-negative staphylococci, two positive blood cultures were required for diagnosis); ii. probable sepsis (negative cultures; ≥3 abnormal findings); iii. possible infection (negative cultures; two abnormal findings); and vi. unlikely infection (negative cultures; single abnormal finding). The following factors were considered[4]: i. maternal risk factors (maternal group B streptococcal positivity, premature rupture of membrane (PROM) >18 h, and chorioamnionitis) for EOS, indwelling central venous or umbilical catheter, ventilator treatment, and parenteral nutrition for LOS; ii. clinical signs of sepsis (respiratory distress, tachycardia/bradycardia, arterial hypotension/poor perfusion, seizures/irritability, lethargy, and ileus); and iii. conventional laboratory tests (abnormal CRP and PCT values). Possible infection and unlikely infection were commonly seen as uninfected[4]. To revise the previous RRs for neutrophils, the “normal” VLBW infants were selected from the unlikely infection group. Those without exposure to prenatal, intrapartum, and postnatal factors[7, 8] known to affect neutrophil values were designated as “normal” VLBW infants. All CBCs from “normal” neonates were collected.

### Statistical analyses

The chart for ATN was constructed by calculating the 5^th^ and 95^th^ percentile for postnatal hours, and the charts for ATI and I:T were constructed by calculating only the 95^th^ percentile because of no lower limit for them. The ATN, ATI, and I:T values were respectively assessed using the revised RRs and those previously reported by Mouzinho[7] and Schmutz[8]. The abnormality of the neutrophil parameters was defined when the neutrophil values were outside the limits of respective RRs. Differences in the ability of the RRs to detect neonates with sepsis were statistically compared using the McNemar’s test. Data also were analyzed in an organism-specific manner for neonates with sepsis attributable to Gram-positive organisms, Gram-negative organisms, and fungi. Sensitivity was defined as the percentage of neonates with proven sepsis who had abnormal neutrophil parameter values. Specificity was defined as the percentage of uninfected neonates with normal neutrophil parameter values. The Youden’s index was calculated on the basis of sensitivity and specificity. Positive predictive value (PPV) and negative predictive value (NPV) were also reported by comparing proven sepsis with uninfected neonates. For neonates with more than one episodes of sepsis, only the first episodes were analyzed. Sensitivity and specificity were compared using the McNemar’s test. PPV and NPV were compared using the *χ*^2^ test. Demographics and clinical variables were analyzed using ANOVA or the *χ*^2^ test. A Bonferroni correction was used in pair-wise comparisons when ANOVA was *P <* 0.05. A *P* value <0.05 was considered significant. Statistical analysis was performed using SPSS 19 (SPSS, IL, USA).

## Results

**Fig 1:**
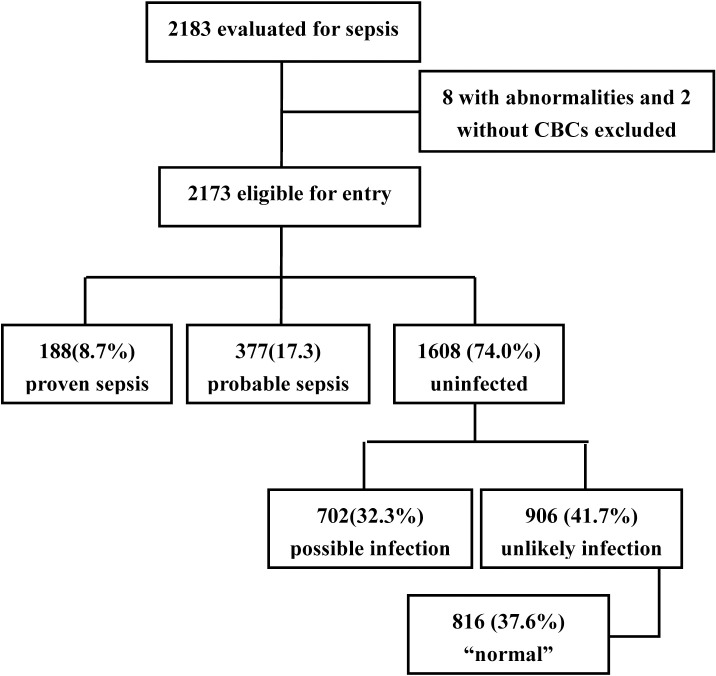
Distribution of neonates with or without sepsis or infection (April 2008 to March 2016).

During the study period, 2183 VLBW infants who admitted to the registered hospitals underwent a comprehensive evaluation for sepsis. Two patients with the diagnosis of congenital malformations and 8 patients without CBC tested were excluded, resulting in 2173 neonates eligible for entry into this study (Fig 1). In total, 188 (8.7%) neonates were classified as proven sepsis, 377 (17.3%) neonates were probable sepsis, and 1608 (74.0%) were uninfected (702 classified as possible infection and 906 as unlikely infection). No differences were observed in maternal variables, neonate’s gestational age, birth weight, gender distribution, or fetal distress. Neonates with proven and probable EOS were more likely delivered by women with PROM, meconium-stained amniotic fluid and maternal chorioamnionitis (Table 1).

**Table 1:** Demographics, clinical variables, and distribution of neonates without and with probable and proven sepsis.

	Proven sepsis		Probable sepsis		Uninfected
	EOS	LOS	EOS	LOS	
Number of infants	31	157	172	205	1608
Gestation age (week)	29.9 ± 1.5	30.0±1.2	30.1±0.8	30.4 ± 1.3	30.3 ± 2.1
Birth weight (g)	1259.2 ± 126.6	1257.3 ± 121.5	1260.9 ± 123.4	1261.6 ± 135.1	1258.5 ± 125.2
Male sex	17 (54.8%)	85 (54.1%)	96 (55.8%)	112 (54.6%)	931 (57.9%)
Cesarean delivery	16 (51.6%)	82 (52.2%)	85 (49.4%)	101 (49.3%)	879 (54.7%)
Resuscitation after delivery	16 (51.6%)	83 (52.9%)	87 (50.6%)	110 (53.7%)	778 (48.4%)
Maternal hypertension	6 (19.4%)	31 (19.7%)	34 (19.8%)	79 (21.0%)	317 (19.7%)
MSAF	9 (29.0%)**△**	13 (8.3%)	45 (26.2%)**△**	18 (8.8%)	140 (8.7%)
Fetal distress	3 (9.7%)	16 (10.2%)	17 (9.9%)	23 (11.2%)	164 (10.2%)
PROM	9 (29.0%)**△**	9 (5.7%)	44 (25.6%)**△**	12 (5.6%)	103 (6.4%)
Chorioamnionitis	8 (25.8%)**△**	6(3.8%)	38 (22.1%)**△**	7 (3.4%)	33 (2.1%)

MSAF: meconium stained amniotic fluid; PROM: premature rupture of membrane

**△***P*<0.001, EOS (proven plus probable) compared with LOS(proven plus probable) or uninfected.

### Revised reference ranges for neutrophils

**Fig 2:**
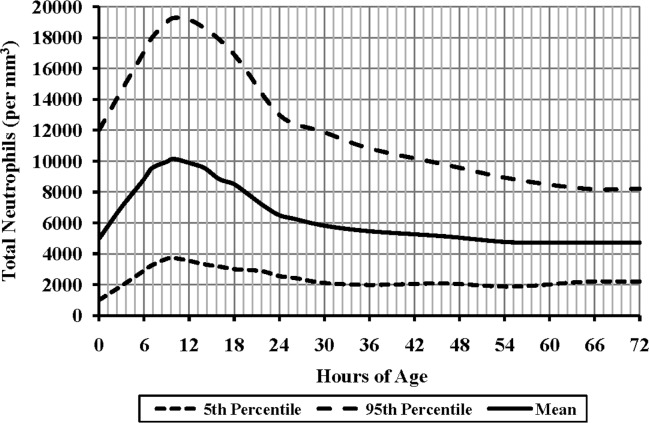
Reference range for absolute total neutrophils (ATN) during the first 72 h after birth of very low birth weight (VLBW) infants. A total of 1472 values were obtained for the analysis. The 5^th^ percentile, 95^th^ percentile, and average are shown.

A total of 816 (37.6%) were designated as “normal” VLBW infants, and 1472 CBCs from 0 to 72 h postnatal age and 3424 CBCs beyond 72 h were obtained. The RR for ATN during the first 72 h is displayed in Fig 2. The peak value was observed at about 10 h of age, with a maximum of 19200 /mm^3^ and a minimum of 3600 /mm^3^. Beyond 72 h, the upper limit for ATN was 8000 /mm^3^ and the lower limit was 2000 /mm^3^.

**Fig 3:**
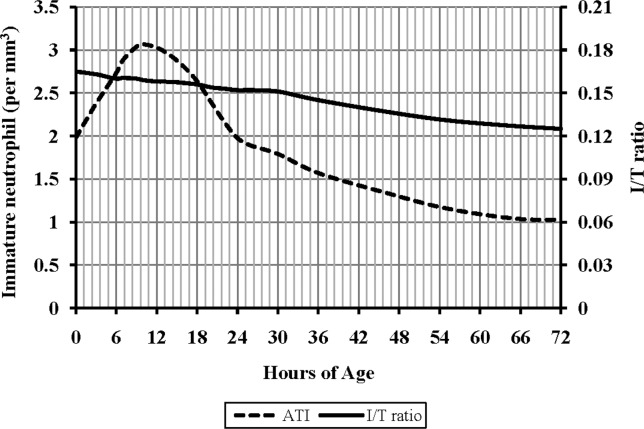
Reference range for the absolute total immature neutrophils (ATI) and immature: total neutrophil proportion (I: T) during the first 72 h after birth of very low birth weight(VLBW) infants. A total of 1472 values were obtained for the analysis. The 95^th^ percentile is shown.

Fig 3 displays the upper limits for ATI and I: T during the first 72 h. The upper limit for ATI was 3000 /mm^3^ at peak and then fell gradually to 1000 /mm^3^ by 72 h. The highest I: T value was generally present closest to delivery (0 h). It then fell gradually to 0.13 by 72 h and remained unchanged afterward.

### Analysis of sensitivity

A total of 188 CBCs were obtained at the time of initial evaluation for sepsis from those neonates who had at least one positive blood culture. The proportion of neutrophil values outside the RRs is presented in Table 2. In episodes of EOS, the elevated I: T and neutropenia were the predominant abnormalities of neutrophil parameters irrespective of the RRs used. In contrast, neutrophilia was significantly more common in episodes of LOS. From 73 to 672 h, the neutrophil values from either the initial or second CBC were more likely to have at least one abnormality using the revised RRs than using Schmutz’s RRs. The difference was significant, and a similar difference in sensitivity was also noted between Mouzinho’s RRs and Schmutz’s RRs. Furthermore, the sensitivity for any abnormality on second CBC at 73–672 h significantly increased compared with that of the initial CBC with either RRs used. However, no significant differences in sensitivity were found for any abnormality on either the initial or second CBC by comparison between revised RRs and Schmutz’s RRs, or between Mouzinho’s RRs and Schmutz’s RRs at 0–72 h. Meanwhile, the comparison of the revised RRs and Mouzinho’s RRs failed to show differential sensitivity both at 0–72 h and at 73–672 h.

**Table 2:** Comparison of the sensitivity of the revised RRs and those of Mouzinho’s RRs and Schmutz’s RRs for neutrophils in VLBW neonates with proven sepsis.

	I:T	Mouzinho				Schmutz				Present			
		↓ATN	↑ATN	ATI	Any*	↓ATN	↑ATN	ATI	Any*	↓ATN	↑ATN	ATI	Any*
All organisms													
0–72 h (31 cases)													
First CBC (31 counts)	45	32	13	35	71	42	3	26	61	55	10	29	77
Second CBC (28 counts)	57	39	18	46	89	54	7	36	79	64	14	39	93
72–672 h (157 cases)													
First CBC (157 counts)	40	5	59	52	82**△**①	7	46	33	67②	11	50	43	79**▲**③
Second CBC (147 counts)	46	10	75	65	95**◇**①	13	63	44	82②	16	67	58	91**◆③**
G+													
73–672 h (115 cases)													
First CBC (115 counts)	36	3	62	54	86**□**④	4	48	36	70⑤	8	52	45	83■⑥
Second CBC (110 counts)	44	9	78	66	97**○**④	12	65	46	83⑤	15	71	62	96**●**⑥
G–													
0–72 h (20 cases)													
First CBC (20 counts)	45	35	10	30	70	45	0	20	60	55⑦	5⑧	25	75
Second CBC(18 counts)	61	39	17	44	83	56	6	28	72	61	11	39	89
73–672 h (41 cases)													
First CBC (41 counts)	44	12	46	41	78	20	32	27	66	27⑦	34⑧	37	78
Second CBC (39 counts)	59	18	72	51	92	26	46	36	80	38	54	51	90
Fungi													
73–672 h (10 cases)													
First CBC (10 counts)	40	0	60	50	90	0	40	40	70	10	50	40	80
Second CBC (8 counts)	50	0	88	75	100	0	75	50	88	13	75	75	100

Values are the percentage of counts outside the reference range.

* indicates that at least one abnormal value was present among I: T, ATN, and ATI values.

**▲***P* = 0.022; **◆***P* = 0.026; **■***P* = 0.030;**●***P* = 0.004, Revised RRs compared with Schumutz’s RRs.

**△***P* = 0.002; **◇***P* <0.001; **□***P* = 0.004;**○***P* <0.001, Mouzinho’s RRs compared with Schmutz’s RRs.

①*P* <0.001; ②*P* = 0.004; ③*P* = 0.004; ④*P* = 0.003; ⑤*P* = 0.028; ⑥*P* = 0.003, the second CBC compared with the first CBC.

⑦*P* = 0.047;⑧*P* = 0.013, late onset episodes compared with early onset episodes in Gram-negative bacteria sepsis.

A total of 206 organisms were isolated from the blood samples of neonates with proven sepsis, including coagulase-negative staphylococcus, *Klebsiella pneumoniae*, *Escherichia coli*, *Enterococcus*, *Candida* species, Group B streptococcus, *Enterobacter cloacae*, *Staphylococcus aureus*, *Pseudomonas aeruginosa*, and *Listeria*. In 18 neonates, 2 pathogens were isolated. The organisms could be categorized into 3 major groups: Gram-positive bacteria (134, 65.0%), Gram-negative bacteria (61, 29.6%), and fungi (11, 5.3%). Because most sepsis (115/134, 85.8%) attributable to Gram-positive bacteria occurred at 73–672 h, data were reported only on these cases. With the revised RRs, at least one neutrophil value was abnormal in 82.5% and 95.5% of the initial and second CBCs, respectively, compared with 69.6% and 82.7%, respectively, using Schmutz’s RRs. The differences were significant, and similar differences were also noted by comparing Mouzinho’s RRs and Schmutz’s RRs. Meanwhile, the second CBC significantly increased the sensitivity for any abnormality compared with that of the initial CBC with either RRs used (*P <* 0.05 for each RRs). As for Gram-negative bacteria sepsis, the occurrence of neutropenia in neonates with early-onset episodes was more common than those with late-onset episodes irrespective of the RRs used, but neutrophilia was significantly more common in late-onset episodes. However, no difference was observed in the sensitivities for any abnormality on either the initial or second CBC between early-onset episodes and late-onset episodes, regardless of the RRs used (*P >* 0.05 for either the initial or second CBC using each RRs). Pair-wise comparison of the three sets of RRs also failed to show differential sensitivity for any abnormality on either the initial or second CBC (*P >* 0.05 for either the initial or second CBC). A total of 11 fungi were isolated, and only 1 case occurred in the first 72 h. Therefore, data were reported only for the 10 late-onset fungal sepsis. Between 73 and 672 h, nearly no neutropenia was found in fungal sepsis for initial and second CBCs, even using the revised RRs, only 1 case showed neutropenia. The percentage of any abnormal value on second CBC test was 100% using both the revised RRs and Mouzinho’s RRs.

### Analysis of specificity, Youden’s index, PPV, and NPV

To determine the predictability of each neutrophil parameter for the presence and/or absence of sepsis, the specificity, Youden’s index, PPV, and NPV for each neutrophil parameter was calculated by comparing proven sepsis with uninfected neonates. The results based on the analysis of the initial CBCs are present in Table 3. In the first 72 h of life, the specificity of ATI and I:T was significantly higher than that of ATN with either RR used (*P <* 0.05 for each RR). When the CBC data were analyzed using the presence of at least one abnormality, the specificity using the revised RRs was significantly higher than that using Mouzinho’s RRs (73% vs 64%, *P <* 0.001), but lower than that using Schmutz’s RRs (73% *vs* 78%, *P <* 0.001). The PPV was poor, regardless of the RR used. However, notably, the NPV ranged from 98% to 99%.

**Table 3:** Comparison of specificity and predictive values of the revised RRs and those of Mouzinho’s RRs and Schmutz’s RRs for neutrophil parameter values by comparing uninfected with proven sepsis in VLBW neonates.

	0–72 h				73–672 h			
	Spec%	Youden's index	PPV	NPV	Spec%	Youden's index	PPV	NPV
ATN								
Mouzinho	78	23	6	98	73	37	37	89
Schmutz	80	25	7	98	81	34	41	88
Present	78	42	9	99	77	38	39	89
ATI								
Mouzinho	85	21	7	98	77	29	35	87
Schmutz	91	17	8	97	84	17	33	84
Present	90	19	9	98	82	25	37	86
I:T								
	89	35	12	98	93	33	58	86
Any*								
Mouzinho	64	35	6	99	62	44	34	93**■**
Schmutz	78	32	7	98	72	38	36	90**■**
Present	73▲	51	9	99	68◆●	47	38	93**■**

Values are the percentage.

*Any abnormality in neutrophil parameters was considered as abnormal, and none of the abnormality seen as normal.

▲*P* <0.001, Revised RRs compared with Mouzinho or Schumutz.

◆*P* = 0.019, Revised RRs compared with Mouzinho.

●*P* <0.001, Revised RRs compared with Schmutz.

**■***P* <0.001, compared with those at 0-72h for each RRs.

From 73 to 672 h, I:T had the greatest specificity compared with ATN and ATI (*P <* 0.05 for each RR). The specificity for any abnormality using the revised RRs was significantly higher than that using Mouzinho’s RRs (68% vs 62%, *P* = 0.019), but lower than that using Schmutz’s RRs (68% vs 72%, *P <* 0.001). Meanwhile, the revised RRs had the highest Youden’s index compared with the previous RRs. The PPV values were modest to poor. Pair-wise comparison of the three sets of RRs failed to show differential NPV, which was much lower than those observed at 0-72h. When any abnormality from both the initial and second CBCs were considered, NPV significantly increased from 94% to 98% using Mouzinho’s RRs, from 90% to 94% using Schmutz’s RRs, and from 93% to 97% using revised RRs (*P* < 0.05 for each RR).

## Discussion

In this multicenter study, altitude-appropriate RRs for neutrophil parameters were constructed from the VLBW infants in Chongqing in southwest of China, where the altitude ranges from 1000 to 2600 feet. Furthermore, the study aimed to find the differences in the usefulness of the altitude appropriate RRs and the previous RRs reported by Mouzinho[7] and Schmutz[8] in evaluation for sepsis in the VLBW infants.

Both the present and the previous RRs showed a transient increase in ATN in the first 24 h. It could be partially explained by the increasing catecholamine concentration after birth[14]. Epinephrine in blood circulation stimulates demargination of neutrophils, which leads to a transient increase in circulating ATN[15]. Generally, neutrophil demargination occurred within minutes of epinephrine injection, and remargination occurred in an hour or so[16]. However, the dynamic changes in ATN did not strictly comply with the epinephrine-induced demargination model because the return of ATN to baseline occurred several days later. Perhaps a longer period of neutrophil demargination due to several hours of epinephrine release was responsible for this peak.

The ATN differences between the present finding and those reported by Mouzinho[7] and Schmutz[8] are listed in Table 4. All values were not strictly comparable, as the gestational age and birth weight was not equal between the studies. The threshold value for defining neutrophilia in this study was much higher than that in Mouzinho’s study[7], but lower than that in Schmutz’s study[8] at peak and after 72 h. This might have been anticipated, as previous reports had revealed a positive correlation between neutrophil concentration and altitude[8, 10, 11]. The city of Dallas where Mouzinho and colleagues carried out their study is near the sea level and Ogden city where Schmutz and colleagues worked is 4800 feet above the sea level. Meanwhile, Chongqing is somewhere in between, with altitude from 1000 to 2600 feet. Neonates born in places with a higher altitude had a higher value of ATN. The reduced oxygen delivery to the fetus in a higher-altitude area may be the underlying mechanism. Thake[17] found that hypoxia equivalent to 4000 meters above the sea level could induce a different response in peripheral venous blood neutrophils, resulting in neutrophilia. The elevated concentration of neutrophils was also noted in rats with exposure to hypoxia in the study by Radom-Aizik [18]. Hypoxia is known to activate inflammation processes in the body[18, 19], and the increased level of inflammatory cytokines, such as interleukin-6, could mobilize neutrophils from the marginated pool into the circulating pool and accelerate the release of neutrophils from the postmitotic pool in the bone marrow, resulting in neutrophilia[20]. However, the comparison of lower limits for ATN did not strictly comply with the altitude difference as shown in Table 4. It may be largely due to that the cohort of VLBW infants in Schmutz’s study was divided into the 28~36 gestation weeks and the lower than 28 gestational weeks when describing the RRs charts. The upper limits for ATI in this study were found to be higher than that in the study by Mouzinho[7] and lower than that in the study by Schmutz [8], however, I:T limits were similar to those in the previous reports[7].

**Table 4:** Comparison of the present ATN reference range with Mouzinho’s chart and Schmutz’s chart.

	Altitude	Gestational	Birth weight (g)	Upper limit (per mm^3^)		Lower limit (per mm^3^)		Time of	Time of
	(feet)	age (week)		At peak	≥72 h	At peak	≥ 72 h	peak (h)	stabilization(h)
Mouzinho	500	29.5 ± 2.6	1157 ± 236	14000	6000	2200	1100	18–20	60
Schmutz	4800	28~36	Unknown	25000	12500	3500	1000	6	72
		<28	Unknown	41000	15300	1500	1300	24	72
Present	1000~2600	30.2 ± 1.9	1258 ± 192	19200	8000	3600	2000	10	60

ATN: absolute total neutrophil.

One objective of the present study was to examine the usefulness of the altitude appropriate RRs for neutrophil parameters in confirming the diagnosis of sepsis in VLBW infants. The incidence of EOS and LOS in this study was 1.4% (31/2173) and 7.2% (157/2173), respectively. The incidence of LOS in this study was lower than that in other studies [1, 21, 22], which may be attributable to the higher birth weight in this study population. Some investigators have shown that neutropenia and elevated I:T predict infection[23]. In the present study, 17 (54.8%) neonates with neutropenia were detected from 31 neonates with proven EOS. Christensen[24] suggested that neutropenia is likely attributable to bone marrow depletion as well as a diminished capacity for accelerated neutrophil production by the preterm, VLBW infants. However, this appears to be limited to the several days after birth[7] because neutrophilia and elevated ATI were commonly observed in the episodes of LOS. Meanwhile, neutropenia was observed more commonly in VLBW infants with Gram-negative sepsis than in neonates with Gram-positive bacterial and fungal sepsis. And among the neonates with Gram-negative sepsis, the neonates with EOS episodes were more likely to show neutropenia than those with LOS. The reasons for these differences are not known, but it may be the combined result of virulence of the infecting organism and the time-dependent hematopoiesis process. Furthermore, 184 of 961 (19.1%) uninfected neonates had neutropenia in this study. This was much higher than that reported by Maheshwari[25], where 8% of all neonates admitted into the NICU had neutropenia. It has been suggested that low gestational age and low birth weight strongly correlate with the incidence of neutropenia in neonates, mainly due to the age-dependent maturation of the immune system and/or nutrient deprivation[2].

I:T is another widely used potential marker of infection. Elevated I:T occurred in less than half of neonates with proven sepsis when only the initial CBCs were considered in this study, which was similar to that reported by Engle[26]. However, I:T was found to have high specificity and high NPV. Its PPV was also significantly higher than ATN and ATI, regardless of the RR used (*P <* 0.05 for each RR). Bender confirmed that this “old” marker of infection was almost as efficient as interleukin-6[27]. However, it was worth noting that when one often depends on a “shift to the left” to identify or exclude infected neonates, the identification of immature neutrophils is problematic, which may limit the use of I:T as a defining criterion[28]. In addition, the present study and Mouzinho’s charts all revealed that the I:T value changed dynamically during the neonatal period, particularly within the first 72 h of life. Therefore, using an invariable threshold of I:T to indentify or exclude the infection in neonates is inappropriate. The present study suggested to define the “elevated I:T” on the basis of I:T value outside the upper limits.

This study indicated that each neutrophil parameter, when used singly, had low sensitivity and PPV, but high specificity and NPV, consistent with the findings of Hornik’s study[29, 30]. In clinical practice, multiple tests comprising these neutrophil parameters are used more commonly. From 0 to 72 h, no significant differences in sensitivity for any abnormality were observed among the three sets of RRs, however, the specificity significantly differed in their pair-wise comparison. VLBW infants often began antibiotic therapy regardless of laboratory values. So, the difference in sensitivity might be of limited clinical significance[26]. However, because of a high NPV, antibiotics should be discontinued if normal serial neutrophil values are observed. So, the striking difference in specificity may be clinically relevant in determining the length of antimicrobial therapy in neonates whose cultures remain sterile[26]. For example, in this study, 913 uninfected neonates underwent the second CBC test: 331 (1: specificity%, 36.3%) neonates were found to have at least 1 abnormal neutrophil value using Mouzinho’s RRs, 203 (1: specificity%, 22.2%) neonates had any abnormality using Schmutz’s RRs, and 239 (1: specificity%, 26.2%) neonates had any abnormality using the revised RRs. It means that when evaluating for sepsis using Mouzinho’s RRs compared with the revised RRs, another 92 neonates would receive unnecessary prolonged antibiotic treatment, which could increase the rates of necrotizing enterocolitis and death in VLBW infants[31].

LOS, compared with EOS, which is mostly caused by vertical transmission, is strongly associated with nosocomial infection and community-acquired infection. The clinical presentation of LOS in neonates is often subtle, so the identification of LOS mainly depends on the laboratory test[1]. Low sensitivity would lead to delayed administration of antibiotics and increased risk for complications, even death. In this study, the sensitivity for any abnormality using Schmutz’s RRs was significantly lower than that using Mouzinho’s RRs and the revised RRs, which meant that 19 out of 157 neonates with proven LOS would have delayed antibiotic treatment when evaluating for sepsis using Schmutz’s RRs compared with the revised RRs. The differences in specificity at 73–672 h were similar to those at 0–72 h, while much lower NPVs were observed at 73–672 hours regardless of the RRs used. However, the second CBC significantly increased the NPV, demonstrating the value of the sequential CBC determination.

The foremost limitation of this study was the retrospective characteristic of this study. Another limitation was the ethnic difference among the three sets of study. The ethnic difference may affect neutrophil values, as Lim[32] reported that different ethnic group had different neutrophil values and the black African people had lower neutrophils than other ethnical groups. But Chan[33] found that the lower neutrophil count which has been observed in adults of African was not present in neonates, and no ethnic differences were found in neutrophil in neonates. Therefore, the ethnicity as an interference factor on neutrophl values could be ignored in this study. Meanwhile, the infant’s sex can also affect the neutrophil counts[8]. Fortunately, no significant difference was found in the sex distribution among the three sets of RRs (the males’ proportion was 49% in Mouzinho’s study, 57.0% in Schmutz’s study, and 57.1% in the present study, *P* = 0.455).

## Conclusions

Although a single neutrophil value has no sufficient power for identifying neonates with either EOS or LOS, multiple tests comprising neutrophil parameters were demonstrated to be useful in diagnosing sepsis in VLBW infants. Sequential CBCs could increase the sensitivity and NPV when any abnormality in neutrophil values was considered. Also, discontinuation of antibiotics is appropriate when sequential CBCs are normal because of the greatest NPV. The revised RRs can be used to better assess circulating neutrophil values in VLBW infants, generally by increasing sensitivity at 73–672 h compared with Schmutz’s RRs and increasing specificity through the entire neonatal period compared with Mouzinho’s RRs. It is concluded that altitude-appropriate RRs for neutrophil parameters in VLBW infants originating from the analysis of a large neonatal database is more suitable to guide the diagnosis of neonatal sepsis.

## Supporting information

S1 STROBE Checklist STROBE checklist in this study. (DOCX)
